# Exploring the Substitution of Fe(III) by Gd(III) in Nanomagnetite

**DOI:** 10.1021/acsnanoscienceau.4c00032

**Published:** 2024-09-03

**Authors:** Carolina Guida, Anthony Chappaz, Agnieszka Poulain, Jean-Marc Grenèche, Alexandre Gloter, Nicolas Menguy, Nathaniel Findling, Laurent Charlet

**Affiliations:** a ISTerre, Univ. Grenoble Alpes, Univ. Savoie Mont Blanc, CNRS, IRD, Univ. Gustave Eiffel, Grenoble 38058, France; b STARLAB, Dept. of Earth & Atmospheric Sciences, Central Michigan University, Mount Pleasant, Michigan 48859, United States; c Grupo geología médica y forense, Universidad Nacional de Colombia, Bogotá 111321, Colombia; d Institut des Molécules et Matériaux du Mans, IMMM UMR 6283, Le Mans Université, Le Mans 72085, France; e Laboratoire de Physique des Solides, Université Paris-Saclay, CNRS UMR 8502, Orsay 91405, France; f Sorbonne Université, Muséum National d’Histoire Naturelle, IRD, Institut de Minéralogie, de Physique des Matériaux et de Cosmochimie (IMPMC UMR CNRS 7590), Paris 75005, France

**Keywords:** Magnetite, Gadolinium, Emerging Contaminant, SPION MRI, Medical Applications

## Abstract

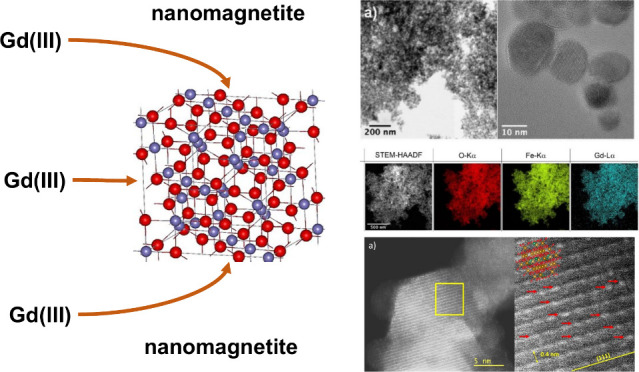

A promising superparamagnetic nanomagnetite dipped with Gd was synthesized for possible medical applications. Its size and morphology are independent of Gd content ranging from 1 to 5%. Gadolinium (III) replaced Fe(III) in the lattice. The sizes of Gd-doped nanoparticles ranged from 5 to 50 nm and exhibited a pure magnetite mineralogical phase.

Gadolinium-based contrast agents (GBCAs) have been widely used in medical magnetic resonance imaging (MRI) since the 1980s.^1^ This generation of Gd complexes is highly stable and inert regarding human biochemistry. However, their environmental impact might not be negligible as originally thought. Increasing levels of Gd have been reported in aquatic systems near major urban centers where several hospitals are present.^2−4^ The San Francisco Bay water experienced an ∼70% increase in Gd concentration over an ∼8-year time period.^5^ The amount of Gd released in the environment for the United States and in the EU was estimated to be ∼21 tons/year and ∼19 tons/year, respectively.^6^ It is expected that, due to the exponential growth of medical applications, the concentration of anthropogenic Gd in aquatic systems will keep increasing.^7,8^

Magnetite is widely used as a powerful remediation agent due to its low production cost, redox-active behavior, magnetic properties, and capability to bind contaminants efficiently.^9,10^ Previous studies have shown that the incorporation of trace metals, such as Co(II), Zn(II), Cr(III), within the crystal lattice of magnetite is possible.^11,12^ A very limited number of studies examined the processes leading to the incorporation of Gd into nanomagnetite.^12,13^ We still ignore whether Gd can be incorporated into the magnetite structure or directly adsorbed on its surface. This crucial information is required for appropriately designing sustainable and efficient solutions preventing the release of Gd into the environment. Additionally, the influence of Gd substitution within magnetite must be refined, because upon oxidation magnetite can be transformed into maghemite. This iron oxide mineral is less interesting for industrial applications, as its reactivity and magnetic susceptibility are lower compared to magnetite.^14,15^

Our objectives were (i) to synthesize Gd-doped magnetite/maghemite nanoparticles and substitute Fe(III) by Gd(III) across a range of loadings (1 to 12% of Gd), (ii) to characterize all the experimental products with an array of different techniques, and (iii) to assess the influence of Gd on nanomagnetite.

The magnetite oxidation into maghemite results from the acidic conditions that favor the release of Fe(II) into solution (eq 1).^16,17^ An inverse linear relationship is reported for Fe concentration in log scale versus pH for values ranging from 5 to 10 (Figure S1).
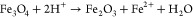
1Assuming that eq 1 is the only process releasing Fe(II) in solution, the percentage of magnetite transformed into maghemite can be determined. For pH values ≥ 7, the magnetite conversion is stalled, and the amount of released Fe(II) remained below 0.07 mM throughout the experiment. At pH 6 (human blood typically has a pH between 7.35 and 7.45), only ∼6% of maghemite was produced; but for lower pH (≤5), the majority of the magnetite was transformed into maghemite, up to ∼82% at pH 3 (Figure S1). The specific surface areas (SSAs) measured directly after synthesizing the nanomagnetites at pH 6 and 8 ranged from 62 to 70 m^2^/g. At pH 4, the SSA increased to 92 m^2^/g.

For all doped nanomagnetites, no detectable levels of Gd were found in the supernatants after solid separation. The concentrations of Gd in nanomagnetites were found to be close to the predicted values (±10%), confirming that Gd was completely fixed by the nanomagnetites.

All nanomagnetite particles exhibit a large distribution in size, ranging from 5 to 50 nm (Figure 1). By applying selected area electron diffraction and high-resolution transmission electron microscopy analyses, we confirmed the presence of magnetite (Figure S2). No differences were observed in the size distribution and shape of nanomagnetites stabilized at pH 4 and 8 and for nanomagnetites coprecipitated with Gd(III). The only noticeable difference was found for particles with 7 and 9% of Gd(III), where some eleongated crystals of goethite measuring between 50 and 100 nm were observed.

**Figure 1:**
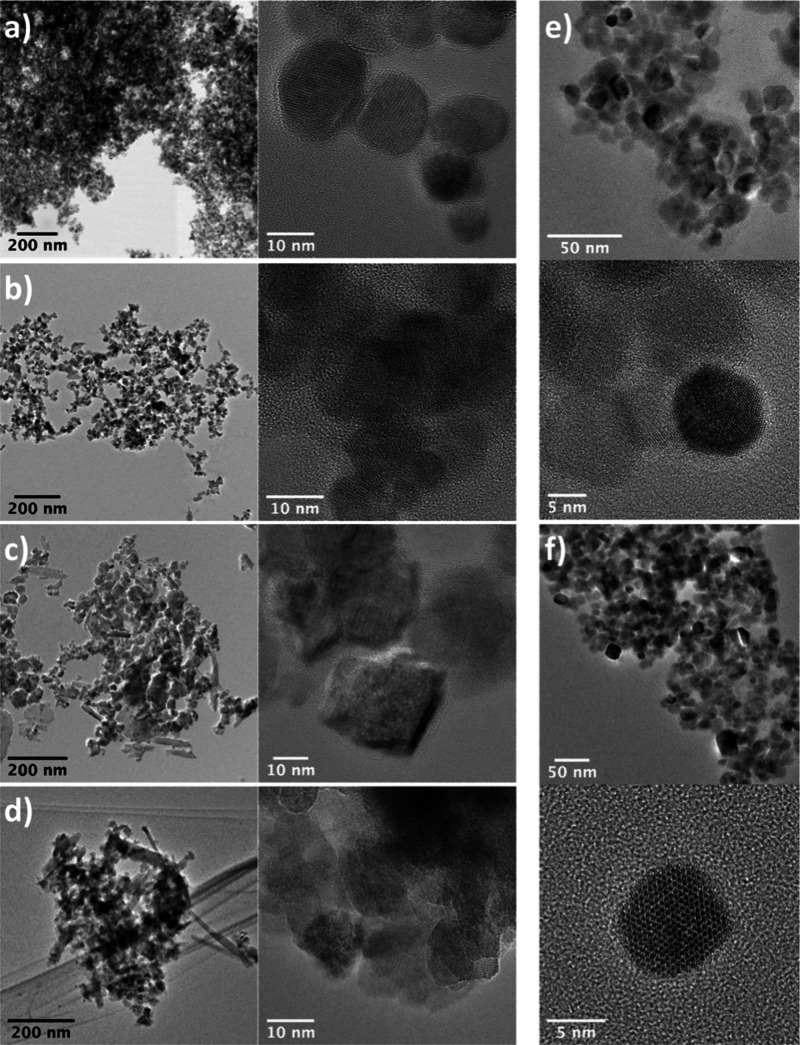
TEM bright field images of nanomagnetites doped with 3% (a), 5% (b), 7% (c), and 9% (d) Gd(III). Panels (e) and (f) show magnetite stabilized at pH = 4 and pH = 8, respectively.

Gadolinium was present and evenly distributed in all nanomagnetites (Figure 2). Additionally, XEDS measurements confirm the Gd concentration within all nanomagnetites increased with the percentage of Gd(III) added during the synthesis (Figure S3).

**Figure 2:**
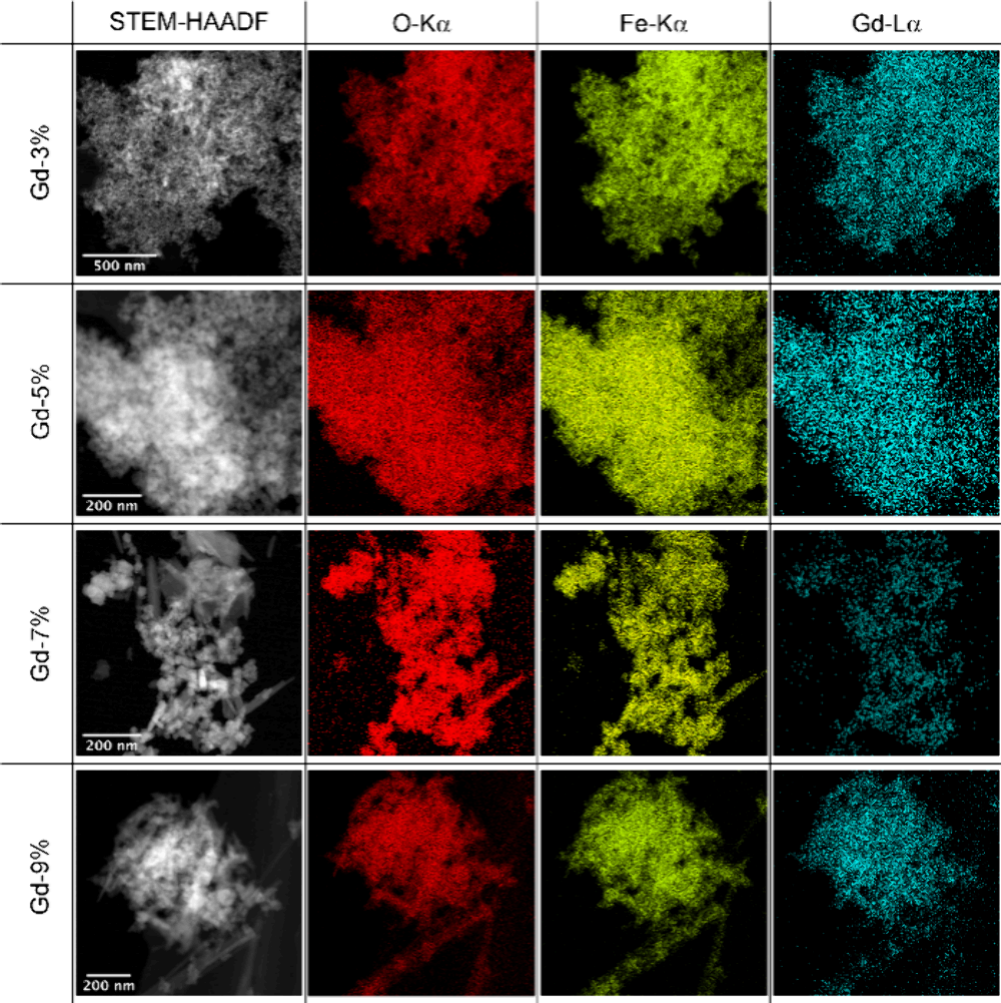
STEM-HAADF and STEM-XEDS elemental mapping of magnetite crystals with 3%; 5%, 7%, and 9% Gd(III).

Here, we used Cs corrected STEM to enhance the localization of Gd in coprecipitated samples. The images captured in pure nanomagnetites reveal contrasting features between columns with varying iron density, such as distinct brightly colored lines formed by iron in octahedral sites on crystals oriented to expose the (111) crystallographic planes. This technique facilitates clear differentiation between Gd and Fe atoms based on their distinct atomic numbers. Whether Gd is situated on the surface or within the structure becomes evident. Providing Gd would be adsorbed, the Gd atoms exhibit contrast spots, more visible near the edge or even at the last surface plane of the particles. The contrast of individual Gd atoms is stronger since they are located, by projection, on the thinnest part of the particle. Conversely, if Gd is present within the structure, it should be observable throughout the particle, often with a weaker contrast. Figure 3 (Figure S7 as well) allows for a comparative analysis of the coprecipitated and adsorbed samples, revealing the identification of individual Gd atoms for both cases. As expected, the adsorbed sample predominantly displays Gd atoms at the particle edge, while in the doped sample, Gd atoms are distributed throughout the particle, seemingly aligned with sites corresponding to an octahedral Fe column XRD patterns of a dry, immediately synthesized nanomagnetite show only peaks typical corresponding to pure magnetite phase (Figures S2 and S4) face-centered cubic unit cell parameter *a* = 8.391(1)–8.397(1) Å). The crystallite sizes estimated from the Scherrer equation give values between 12 and 17 nm (Table S1). The width of the diffraction peaks of the three stabilized samples for pH 3, 5, and 8 are similar (Figure S4), excluding a decrease in the magnetite crystal size, in agreement with prior studies.^18^ For a 16 nm spherical particle with 20% unmodified nanomagnetite, we estimated an inner diameter of 9.4 nm and an oxidized layer around the magnetite core of ∼ 3 nm thickness. However, the TEM images (Figure 1f) show a larger size distribution (∼5–50 nm). Therefore, it is possible that some small particles may be completely oxidized, while others may have only a thin layer of oxidation at the surface.

**Figure 3:**
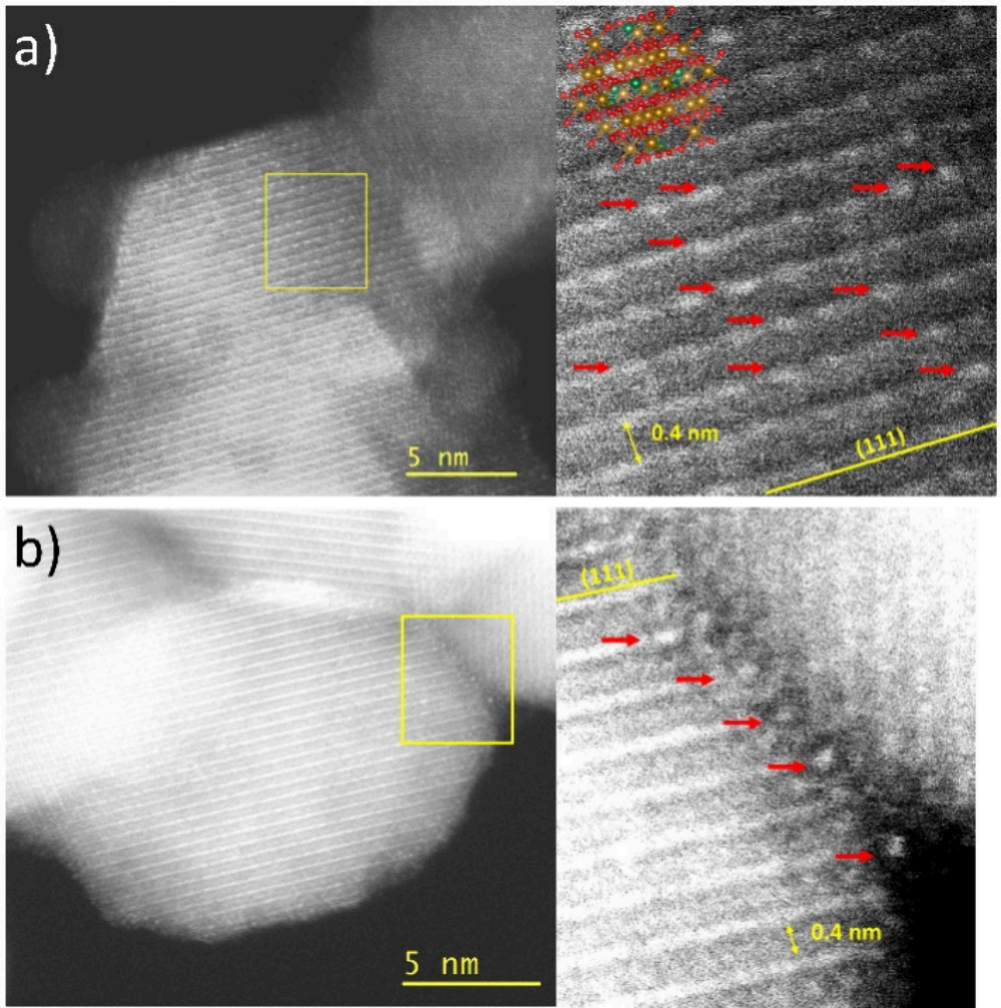
HAADF STEM images of (a) doped nanomagnetite containing 4% Gd (III) and (b) Gd adsorbed on pure nanomagnetite.

The particle size of the Gd-nanomagnetite samples showed no significant difference compared with pure nanomagnetites, supporting our TEM observations. Additionally, no shifted diffraction peak was observed nor was a peak corresponding to a Gd hydroxide phase detected in the diffraction patterns. However, in those samples containing more than 5% Gd(III), the solids showed crystallized phases of goethite. Magnetite doped with 8% Gd seems to contain ∼5% of a green rust phase with a pyroaurite structure. For nanomagnetites coprecipitated with 7% and 9% Gd(III), the presence of less than ∼3% of green rust was less obvious because the XRD detection limit is ∼ 1%.

For magnetite to be formed, the Fe(II)/Fe(II)+Fe(III) ratio must be equal to 0.66 (pH range: 8.5–12; ionic strength: 0.5–3M^16^). Our nanomagnetite synthesis involved a high concentration of OH^–^, which maintained the solution pH close to 12 for all preparations. For solutions containing 1 to 5% Gd(III), the Fe(II)/Fe(II)+Fe(III) ratio varied between 0.655 and 0.667 (Figure. S6). For concentrations between 6 and 12%, the Fe(II)/Fe(II)+Fe(III) ratio ranged from 0.638 to 0.653 (values < 0.66), which is below the stoichiometric value required for the formation of magnetite. As a result, goethite and green rust formed through the dissolution–crystallization process.

A typical Mössbauer spectrum for magnetite at room temperature consists of two sextets: (i) Fe(III) in tetrahedral positions and (ii) Fe(III) and Fe(II) in octahedral positions, that appear as an average Fe^2.5+^ resulting from fast electron exchange. Magnetite spectra collected at T = 77 K, below the Verwey transition (∼119 K) where electron hopping is absent, show the expected structural change from a cubic to a monoclinic system.^19^

For the Gd free nanomagnetite sample (pH = 8; T = 300 and 77 K), typical spectra expected for magnetite were collected. The sextet lines are wider than expected for crystalline magnetite, and the broadening was attributed to the presence of superparamagnetic relaxation effects due to the small size and distribution of particles. The mean value of the isomer shift is typical of the exclusive presence of Fe(III) and, therefore, consistent with the presence of nanomagnetites

For the nanomagnetite samples doped with Gd(III) ranging from 1 to 5%, the spectra at 300 K show broader lines than those for Gd free nanomagnetite, while the hyperfine structures at 77 K are rather similar. These features can be explained both by the presence of superparamagnetic nanoparticles and the additive role of Gd(III) ions introducing internal and/or surface structural disorder.

The magnetite-to-maghemite conversion ratios obtained from Mössbauer results and ICP-AES measurements are similar (see Figure S1) and confirm prior findings.^10^ For Gd-doped nanomagnetites (1 to 5%), the mean isomer shift values are consistent with the oxidation of some Fe species, allowing an estimate of the mean composition of the nanoparticles (Table S2), supported by the XRD data. Although, there were differences observed in the spectra between 300 and 77 K, the cause of this discrepancy remains unknown.

The incorporation of structural Gd is possible if Gd(III) replaces Fe(III) atoms in an octahedral position. Such substitution is possible because the charge and size are similar (0.938 and 0.645 Å for Gd and Fe, respectively). However, Gd(III) displays a much larger ionic radius than Fe(III) that may hinder or condition at low concentrations its incorporation into the magnetite lattice.^20^ This assumption is supported by the analysis of coprecipitated nanomagnetites with 7 and 9% of Gd. The Mössbauer spectra differ significantly from the spectra of Gd free magnetite (pH = 8) (Figure 4). Significant new quadrupolar doublets were observed in the central part (see red curve), suggesting the presence of some Fe-containing impurities. Indeed, the X-ray patterns indicate the presence of other mineral phases, such as goethite and green rust. The first phase generally contributes to a magnetic sextet with broadened lines and a specific value of quadrupolar shift (∼0.20 mm/s) and/or quadrupolar doublet depending on the size of the particles. The complexity of the hyperfine structure prevents the identification and estimation of any goethite content. But the red curve (quadrupolar doublets) could be attributed to some Fe species located in structurally and chemically disturbed green rusts.

**Figure 4:**
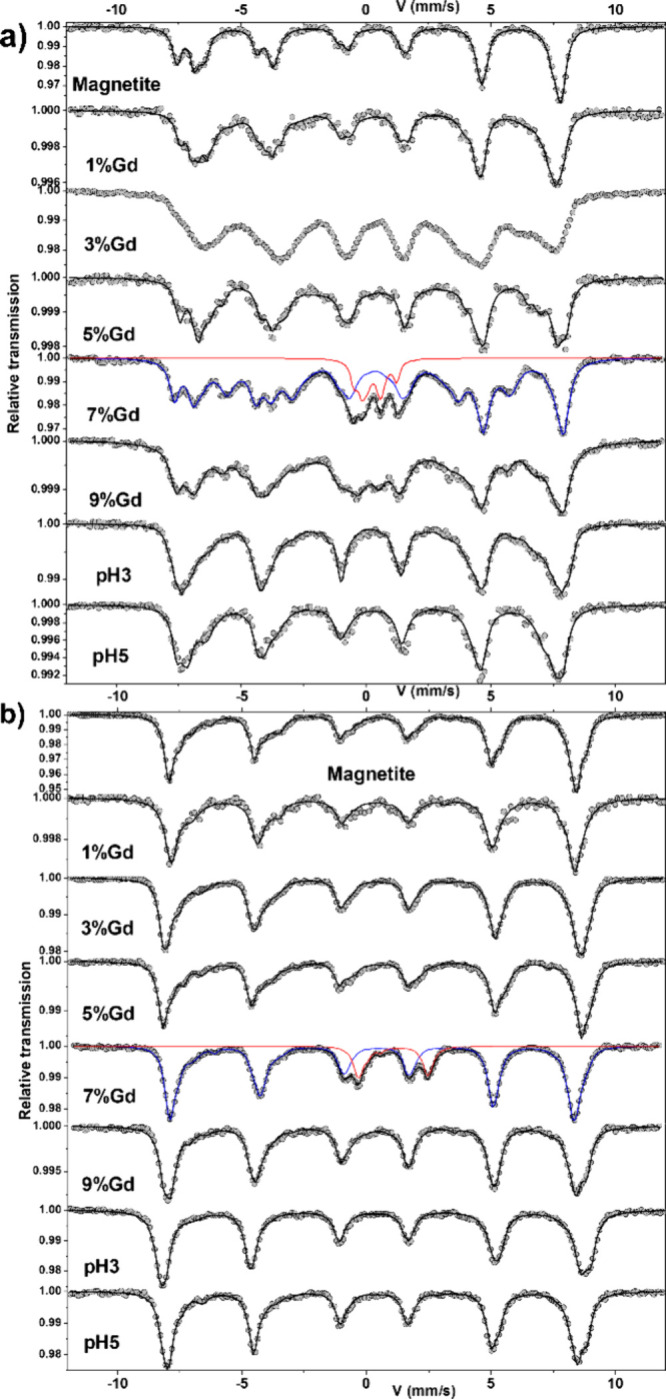
Mössbauer spectra measured at 300 K (a) and 77 K (b) for Gd free nanomagnetite, nanomagnetite equilibrated at pH 3 and 5, and nanomagnetite doped with 1–9% of Gd (III) fitted with different components containing Fe^2.5+^ (red line) and Fe(III) (blue line) species.

Conversely, the hyperfine spectral structures for nanomagnetites at pH = 3 and 5 differ significantly from the previously discussed spectra for Gd-rich magnetites (Figure 4). These differences result from magnetic components with broadened and asymmetric lines that can be described via a linearly correlated distribution of hyperfine fields and isomer shifts. The most relevant parameter is given by the average values of the isomeric shifts, which are intermediate between those typically observed in oxide phases containing Fe(II) and Fe(III) species. Such description is consistent with a mixture of stoichiometric magnetite and maghemite phases (Table S2 and Figure S1;^21,22^) and confirms the absence of maghemite in Gd-rich particles.

We synthesized nanomagnetites with a stoichiometric ferrous-to-ferric ions ratio close to 0.5 and a size ranging from 5 to 50 nm. Our results show that the maghemite level remained below ∼10%, and that Gd(III) is uniformly distributed throughout the nanoparticles in lieu of Fe(III) in octahedral sites. Gd-doped nanomagnetite samples form true Fe_(3-x)_Gd_x_O_4_ solid solutions, 0.02 < x < 0.1, with Fe(III) being substituted by Gd(III) within the magnetite superparamagnetic iron oxide nanoparticles (SPION). Such trapping (or substitution) within the mineral structure prevents the release of Gd(III) and unlocks its potential as a theragnostic agent within the cytoplasm or nucleus of the targeted cells. These nanoparticles clearly present a strong potential for medical applications, combining the advantages of SPION nanoparticles and Gd-concentrates particles for imaging, therapeutic, or theragnostic applications. Moreover, this composite is environmentally friendly as it could be readily eliminated through conventional wastewater treatment methods.

## Supporting Information Available

Materials and methods, calculated magnetite to maghemite data conversion from ICP-AES and Mössbauer spectroscopy, additional magnetite crystal analysis of TEM, SAED, HRTEM and STEM; XRD patterns data from all magnetite synthesis, calculated parameters after Rietveld refinements and a representative Rietveld analysis pattern of powder diffraction data; ratios of iron and OH concentrations used to prepare Gd-doped magnetites and the refined values of the hyperfine parameters of Mössbauer (PDF)

## References

[ref1] Lux J.; Sherry A. D. Advances in Gadolinium-Based MRI Contrast Agent Designs for Monitoring Biological Processes in Vivo. Current Opinion in Chemical Biology 2018, 45, 121–130. 10.1016/j.cbpa.2018.04.006. 29751253 PMC6076858

[ref2] Altomare A. J.; Young N. A.; Beazley M. J. A Preliminary Survey of Anthropogenic Gadolinium in Water and Sediment of a Constructed Wetland. Journal of Environmental Management 2020, 255, 109897 10.1016/j.jenvman.2019.109897. 31783213

[ref3] Rogowska J.; Olkowska E.; Ratajczyk W.; Wolska L. Gadolinium as a New Emerging Contaminant of Aquatic Environments. Environmental toxicology and chemistry 2018, 37 (6), 1523–1534. 10.1002/etc.4116. 29473658

[ref4] Souza L. A.; Pedreira R. M. A.; Miró M.; Hatje V. Evidence of High Bioaccessibility of Gadolinium-Contrast Agents in Natural Waters after Human Oral Uptake. Science of The Total Environment 2021, 793, 148506 10.1016/j.scitotenv.2021.148506. 34182440

[ref5] Hatje V.; Bruland K. W.; Flegal A. R. Increases in Anthropogenic Gadolinium Anomalies and Rare Earth Element Concentrations in San Francisco Bay over a 20 Year Record. Environ. Sci. Technol. 2016, 50 (8), 4159–4168. 10.1021/acs.est.5b04322. 26742888

[ref6] Brünjes R.; Hofmann T. Anthropogenic Gadolinium in Freshwater and Drinking Water Systems. Water Res. 2020, 182, 115966 10.1016/j.watres.2020.115966. 32599421 PMC7256513

[ref7] Kaegi R.; Gogos A.; Voegelin A.; Hug S. J.; Winkel L. H.; Buser A. M.; Berg M. Quantification of Individual Rare Earth Elements from Industrial Sources in Sewage Sludge. Water research X 2021, 11, 100092 10.1016/j.wroa.2021.100092. 33733081 PMC7937830

[ref8] Kümmerer K.; Helmers E. Hospital Effluents as a Source of Gadolinium in the Aquatic Environment. Environmental science & technology 2000, 34 (4), 573–577. 10.1021/es990633h.

[ref9] Cheng W.; Marsac R.; Hanna K. Influence of Magnetite Stoichiometry on the Binding of Emerging Organic Contaminants. Environmental science & technology 2018, 52 (2), 467–473. 10.1021/acs.est.7b04849. 29215874

[ref10] Gorski C. A.; Scherer M. M. Determination of Nanoparticulate Magnetite Stoichiometry by Mossbauer Spectroscopy, Acidic Dissolution, and Powder X-Ray Diffraction: A Critical Review. Am. Mineral. 2010, 95 (7), 1017–1026. 10.2138/am.2010.3435.

[ref11] Li Y.; Wei G.; Liang X.; Zhang C.; Zhu J.; Arai Y. Metal Substitution-Induced Reducing Capacity of Magnetite Coupled with Aqueous Fe (II). ACS Earth and Space Chemistry 2020, 4 (6), 905–911. 10.1021/acsearthspacechem.0c00089.

[ref12] He H.; Zhong Y.; Liang X.; Tan W.; Zhu J.; Yan WANG C. Natural Magnetite: An Efficient Catalyst for the Degradation of Organic Contaminant. Sci Rep 2015, 5 (1), 10139 10.1038/srep10139. 25958854 PMC4426601

[ref13] Janani V.; Induja S.; Jaison D.; Meher Abhinav E.; Mothilal M.; Gopalakrishnan C. Tailoring the Hyperthermia Potential of Magnetite Nanoparticles via Gadolinium ION Substitution. Ceram. Int. 2021, 47 (22), 31399–31406. 10.1016/j.ceramint.2021.08.015.

[ref14] Jordan N.; Ritter A.; Scheinost A. C.; Weiss S.; Schild D.; Hübner R. Selenium(IV) Uptake by Maghemite (γ-Fe _2_ O _3_). Environ. Sci. Technol. 2014, 48 (3), 1665–1674. 10.1021/es4045852. 24422437

[ref15] Rebodos R. L.; Vikesland P. J. Effects of Oxidation on the Magnetization of Nanoparticulate Magnetite. Langmuir 2010, 26 (22), 16745–16753. 10.1021/la102461z. 20879747

[ref16] Jolivet J.-P.; Chanéac C.; Tronc E. Iron Oxide Chemistry. From Molecular Clusters to Extended Solid Networks. Chem. Commun. 2004, (5), 477–483. 10.1039/B304532N. 14973569

[ref17] Peng H.; Pearce C. I.; Huang W.; Zhu Z.; N'Diaye A. T.; Rosso K. M.; Liu J. Reversible Fe(Ii) Uptake/Release by Magnetite Nanoparticles. Environmental Science: Nano 2018, 5 (7), 1545–1555. 10.1039/C8EN00328A.

[ref18] Moon J.-W.; Rawn C. J.; Rondinone A. J.; Wang W.; Vali H.; Yeary L. W.; Love L. J.; Kirkham M. J.; Gu B.; Phelps T. J. Crystallite Sizes and Lattice Parameters of Nano-Biomagnetite Particles. Journal of Nanoscience and Nanotechnology 2010, 10 (12), 8298–8306. 10.1166/jnn.2010.2745. 21121331

[ref19] Doriguetto A. C.; Fernandes N. G.; Persiano A. I. C.; Filho E. N.; Grenèche J. M.; Fabris J. D. Characterization of a Natural Magnetite. Phys Chem Minerals 2003, 30 (5), 249–255. 10.1007/s00269-003-0310-x.

[ref20] Schock H. H. Distribution of Rare-Earth and Other Trace Elements in Magnetites. Chem. Geol. 1979, 26 (1–2), 119–133. 10.1016/0009-2541(79)90034-2.

[ref21] Greneche J.-M. The Contribution of 57 Fe Mössbauer Spectrometry to Investigate Magnetic Nanomaterials. In Mössbauer spectroscopy; Springer, 2013; pp 187–241.

[ref22] Bogart L. K; Fock J.; da Costa G. M; Witte K.; Greneche J.-M.; Zukrowski J.; Sikora M.; Latta D. E; Scherer M. M; Hansen M. F.; Frandsen C.; Pankhurst Q. A Prenormative Verification and Validation of a Protocol for Measuring Magnetite–Maghemite Ratios in Magnetic Nanoparticles. Metrologia 2022, 59 (1), 015001 10.1088/1681-7575/ac36b6.

