# Supplementary material for: Exploring the Substitution of Fe(III) by Gd(III) in Nanomagnetite
Source: ACS Nanosci Au. 2024 Sep 3;4(5):322–6. doi: 10.1021/acsnanoscienceau.4c00032 (PMC11487657; doi:10.1021/acsnanoscienceau.4c00032)
Supplement: Supplementary file 1 — ng4c00032_si_001.pdf [file ng4c00032_si_001.pdf]

Electronic Supplementary Information

Exploring the substitution of Fe(III) by Gd(III) in nanomagnetite

Carolina Guida[a,b,c], Anthony Chappaz[b], Agnieszka Poulain[a], Jean-Marc Grenèche[d], Alexandre Gloter[e], Nicolas Menguy[f], Nathaniel Findling[a], and Laurent Charlet[a]*

[a]Univ. Grenoble Alpes, Univ. Savoie Mont Blanc, CNRS, IRD, Univ. Gustave Eiffel, ISTerre, 38000 Grenoble, France.

[b]STARLAB, Dept. of Earth & Atmospheric Sciences, Central Michigan University, 48859 Michigan, USA.

[c]Grupo geología médica y forense, Universidad Nacional de Colombia, Apartado Aéreo, Bogotá, Colombia.

[d]Institut des Molécules et Matériaux du Mans, (IMMM CNRS UMR 6283), Le Mans Université, F-72085 Le Mans, France.

[e]Laboratoire de Physique des Solides, Université Paris-Saclay, CNRS UMR 8502, 91405, Orsay, France.

[f]Sorbonne Université, Muséum National d'Histoire Naturelle, IRD, Institut de Minéralogie, de Physique des Matériaux et de Cosmochimie (IMPMC UMR CNRS 7590), F-75005, Paris, France.

*Corresponding author: anthony.c@cmich.edu

METHODS

Chemicals

Gadolinium (III) chloride hexahydrate (99.999%), sodium nitrate ACS reagent, ≥ 99.0%, nitric acid, ≥ 65 %, p.a., ISO, hydrazine hydrate (≥ 95%), iron (II) chloride tetrahydrate (99.99%), iron (III) chloride hexahydrate (≥ 99%), and ammonia solution, 25% (99%), were purchased from Sigma-Aldrich. Sodium hydroxide (≥ 99%) was bought from Roth, and hydrochloric acid, 48%, extra pure, SLR, from Fisher Chemical. All solutions were made with deionized Milli-Q water, and all experiments were performed under Ar atmosphere at room temperature within a glovebox. All containers were soaked in 5% HNO_3 for 24h, cleaned for 10 minutes in an ultrasonic bath, and rinsed thoroughly with deionized water before use.

Nanomagnetite and Gd doped nanomagnetite synthesis

Nanomagnetites and Gd doped nanomagnetites were synthesized using the alkalizing aqueous Fe(II) and Fe(III) ions method[1,2]. Briefly, 25 mL stock solutions of 0.8 M $FeCl_2$, 1.6 M $FeCl_3$, 1.6

M $GdCl_3$, and 60 mL of 6 M of NH_4OH were prepared. A drop of hydrazine hydrate was added to the $FeCl_2$ solutions to prevent ferrous oxidation. For co-precipitation experiments, a precise amount of Gd stock solution was added (0.25-1.8 mL) to replace the right amount of Fe(III) in the ferric solution. The Gd percentage calculated to replace Fe(III) ranged from 1 to 12%. As a first step, both ferrous and ferric chloride solutions were mixed. Then, the NH_4OH solution was slowly injected into the ferrous/ferric chloride solution (injection rate: 0.3mL/s) and stirred for 24 hours. Subsequently, the supernatant was separated and acidified with HNO_3 65% prior to ICP-AES measurements. Finally, the suspension was washed by replacing the supernatant with degassed water six times, vacuum filtered with a 0.22 µm filter, and dried under an anoxic atmosphere. Selected Gd-doped nanomagnetite samples were digested with 65% HNO_3 for elemental analysis.

Magnetite oxidation into maghemite

Structural changes and stability of magnetite were investigated across a wide pH range (3-10). Eight experiments were carried out, with the initial concentration of the magnetite fixed at 10 g/L and 0.1 mM NaCl background solution. pH was adjusted using HCl and NaOH solutions until the values remained stable within 0.2 units of pH over a 24 h period. After 3 weeks, a magnet was used to concentrate the magnetic solid at the bottom of the reactors to separate it from the liquid. All liquids were then filtered by a syringe filter (0.22 µm) to determine the iron concentration in the solution. The remaining solids were pre-dried using a vacuum filtration system (0.22 µm) and left overnight in the glovebox for a final drying step.

Elemental analysis

Total Gd and Fe concentrations in the liquid samples were determined by ICP-AES (Varian 720 ES) at wavelengths of 234,350 nm for Fe and 358,496 nm for Gd using external calibration. The RSD was 4% for Fe and 2% for Gd. The limits of detection were 4.96 µM for Fe and 0.05 µM for Gd. The limits of quantification were 15.02 µM for Fe and 0.16 µM for Gd.

Solid characterization

XRD. X-ray powder diffraction measurements were conducted with a diffractometer [CuKα radiation (λ = 1.54 Å); Bruker D8] using a Debye–Scherrer configuration with an elliptical mirror

to obtain a high flux and parallel incident beam, and an a SolX Si(Li) solid-state detector to collect the diffracted beam. The patrons were collected with a step size of 0.026° in the 20° to 70° 2-theta range at room temperature. XRD pattern samples were loaded inside the glovebox into the airtight polymethyl methacrylate sample holder with a transparent dome to avoid any possible oxidation. The lattice parameters and the average crystallite sizes were calculated using the DebyeScherrer equation and the Profex software[3]. The refinement with a free lattice parameter was performed for all samples.

TEM and STEM-EELS. A few milligrams of nanomagnetite samples were placed in plastic vials, filled with 10 mL of ethanol, sealed with parafilm, and removed from the glovebox for 5 minutes for redistribution in an ultrasonic bath. These dilute suspensions were drop-casted on pure carbon, 200 mesh Cu TEM grids and dried. The samples were transferred for TEM and STEM measurement under anoxic conditions and were in contact with air only for a few minutes during mounting on the microscope sample holder. TEM observations (HRTEM), high-angle annular dark field imaging in scanning transmission electron microscope mode (STEM-HAADF) and X-ray energy-dispersive spectroscopy (XEDS) mapping experiments) were performed on a Jeol JEM 2100F microscope, operating at 200 kV, equipped with a Schottky emitter, a Jeol detector with an ultra-thin window allowing detection of light elements and a scanning TEM (STEM) device. Cs corrected STEM-EELS measurements were performed in a NION UltraSTEM200 operated at 100 kV with a spatial resolution at ca. 0.1 nm and coupled with a high-sensitivity EELS spectrometer. Data was collected from clean GR plates; those that were considered free of contamination and/or visible stray iron (oxyhydr)oxide minerals.

BET. The specific surface area (SSA) was determined by the Brunauer−Emmett−Teller adsorption method (BET-N_2) at 77 K, using a Belsorp-Max (Bel Japan) volumetric gas sorption instrument. A small amount (0.418 g) of magnetite was loaded in a glass cell inside the glovebox and then dried under vacuum at 80 ^{0}C for 12 h. The SSA was calculated from the BET equation in the P/P_0 range 0.052 – 0.307.

^{57}Fe Mössbauer spectrometry. Mössbauer spectra were collected at 300 and 77 K using a conventional constant acceleration transmission spectrometer with a ^{57}Co(Rh) source and an α-Fe foil for calibration at room temperature. Samples containing 5 mg of Fe/cm^2 (□ 20 mg of the

solids) were loaded in round plastic holders, sealed in glove box with epoxy glue to prevent possible oxidation, and then transported under oxygen-free conditions. The obtained spectra were fitted with an in-house program (MOSFIT). The description of the hyperfine structures consisted of a least-squares fit, including quadrupolar doublets and Zeeman magnetic sextets composed of Lorentzian lines.

References

1. A. C. Scheinost and L. Charlet: Selenite Reduction by Mackinawite, Magnetite and Siderite: XAS Characterization of Nanosized Redox Products. *Environ. Sci. Technol.* **42**(6), 1984 (2008).
2. J. P. Jolivet, P. Belleville, E. Tronc, and J. Livage: Influence of Fe(II) on the formation of the spinel iron oxide in alkaline medium. *Clays Clay Miner* 531 (1992).
3. N. Doebelin and R. Kleeberg: Profex: a graphical user interface for the Rietveld refinement program BGMN. *Journal of applied crystallography* **48**(5), 1573 (2015).

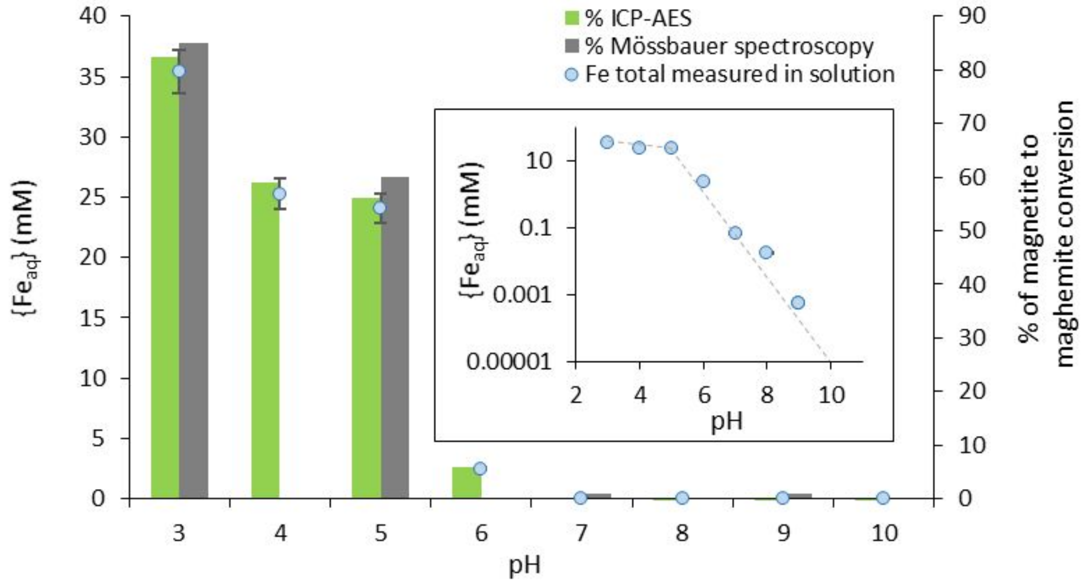

Figure S1. Fe concentration released in solution during 3 weeks of stabilization experiments over a pH range from 3 to 10 (blue dots). The green and gray bars, taken from ICP-AES and Mössbauer spectroscopy respectively, show the calculated magnetite to maghemite conversion. The inset graph plots Fe total measurements on a logarithmic scale.

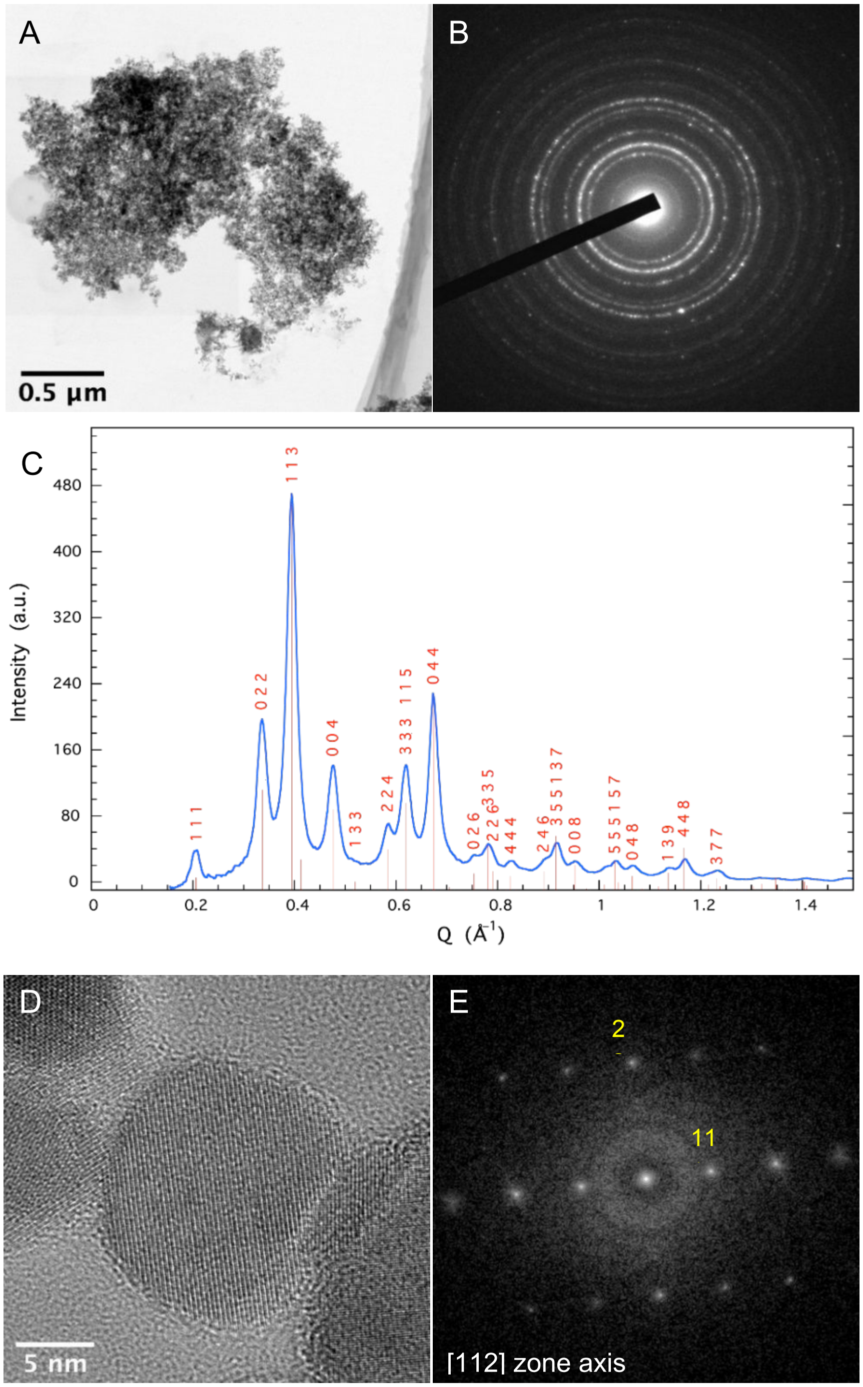

Figure S2. TEM bright field image (A), selected area electron diffraction (B), and corresponding radial intensity (C). High-Resolution TEM image of a magnetite crystal and related fast Fourier transform (E). Diffraction pattern and FFT are indexed with magnetite structural parameters (a = 8.396 Å and Fd3m spacegroup)

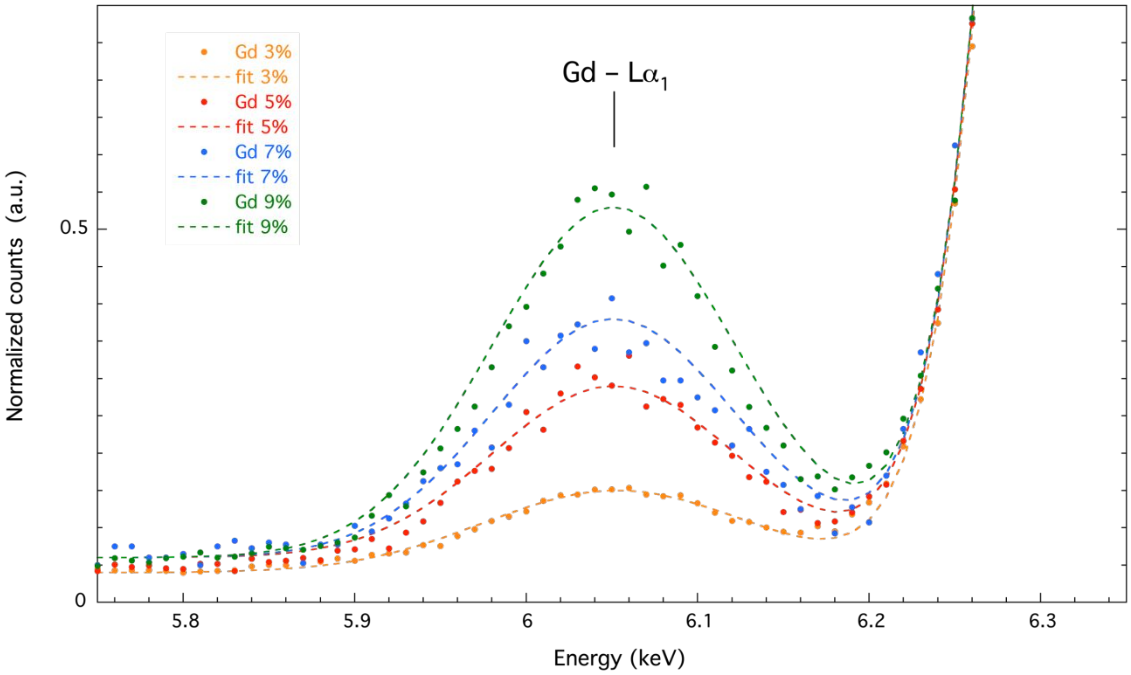

Figure S2. Gadolinium $L\alpha_1$ line profile as a function of Gd-content. Spectra were normalized against the Fe-Kα line.

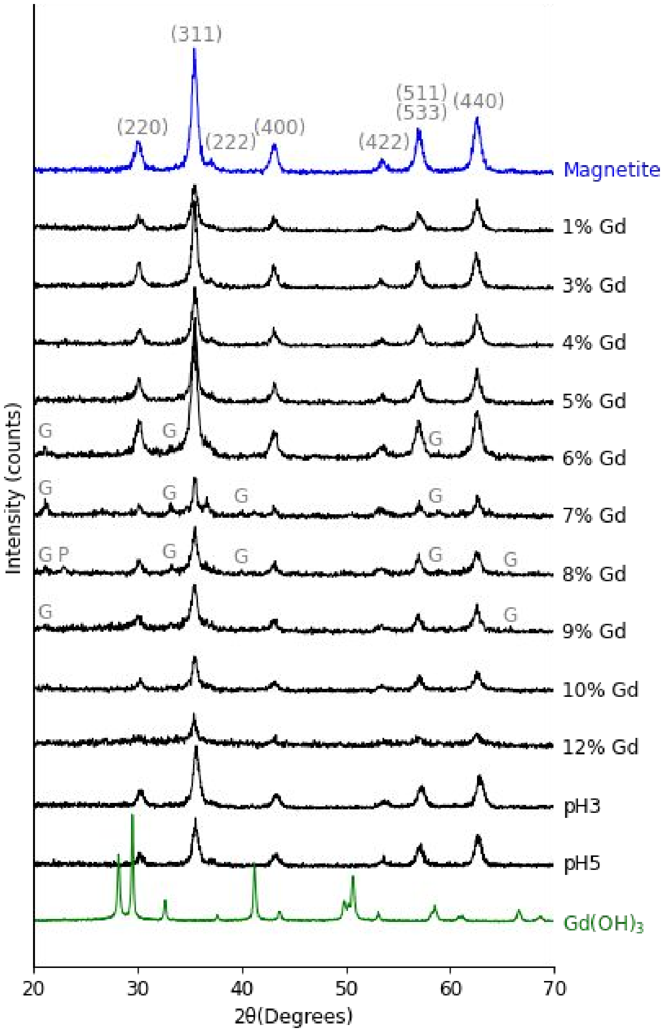

Figure S3. XRD patterns data from all magnetite synthesis. XRD data of magnetite stabilized at pH 8 (blue), pH 3, pH 5 and $Gd(OH)_3$ (at the bottom) and magnetite with 1-12% of Gd(III) as Fe(III). The XRD peaks mainly show magnetite and a minor fraction of goethite (G) and green rust (P) phases in samples The references used correspond to JCPDS PDF 00-019-0629 for magnetite, JCPDS PDF 00-029-0713 for goethite (G), ICSD-80876, and PDF 01-086-0181 for green rust (P).

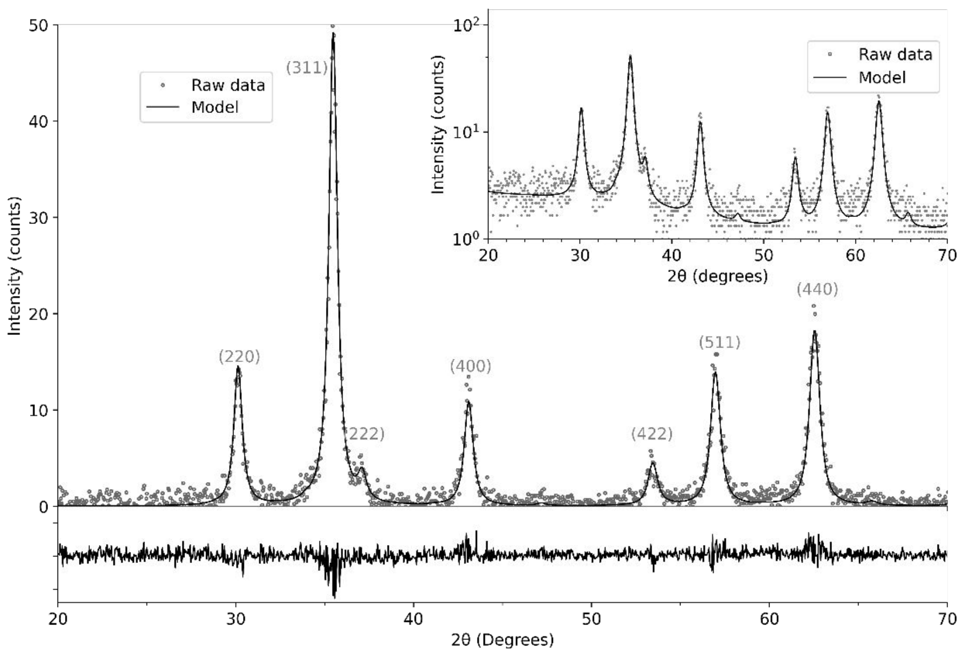

Figure S4. Representative Rietveld analysis pattern of powder diffraction data of magnetite doped with 3% Gd(III) as Fe(III) for only cell parameters determination. The fit is made with magnetite (M) reference lattice parameters (JCPDS PDF 00-019-0629). The residue between the observed and calculated intensities is plotted below the profile. The same data is plotted on a logarithmic scale in the inset graph. Goethite and green rust phases are not present in this sample.

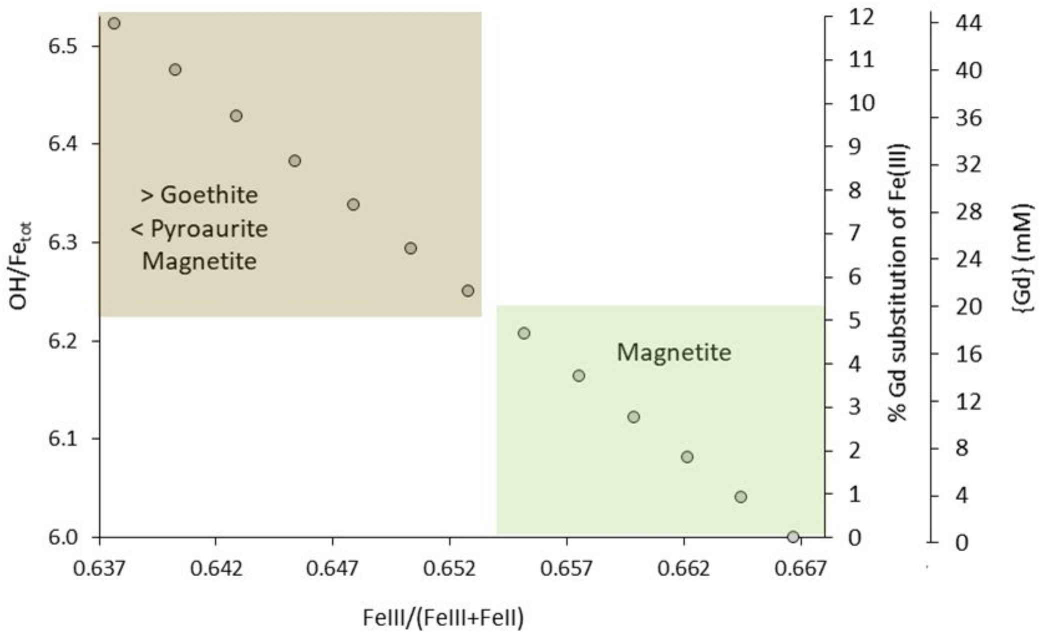

Figure S5. The plot of ratios of iron and OH^- concentrations used to prepare Gd-doped magnetites. OH/Fetotal, Fe(III)/(Fe(III)+Fe(II)) and %Gd(III) substitution of Fe(III) were calculated with 3272.73 mM OH^-, the estimated total Fe concentration for each sample, 181.82 mM Fe(II) and the calculated Fe(III) and Gd(III) values utilized for the coprecipitated sample preparation. The data are grouped into two groups Fe(III)/(Fe(III)+Fe(II)): values lower than 0.665 corresponding to experimental products containing additional phases like goethite and green rust; and higher than 0.665, corresponding to pure nanomagnetite particles.

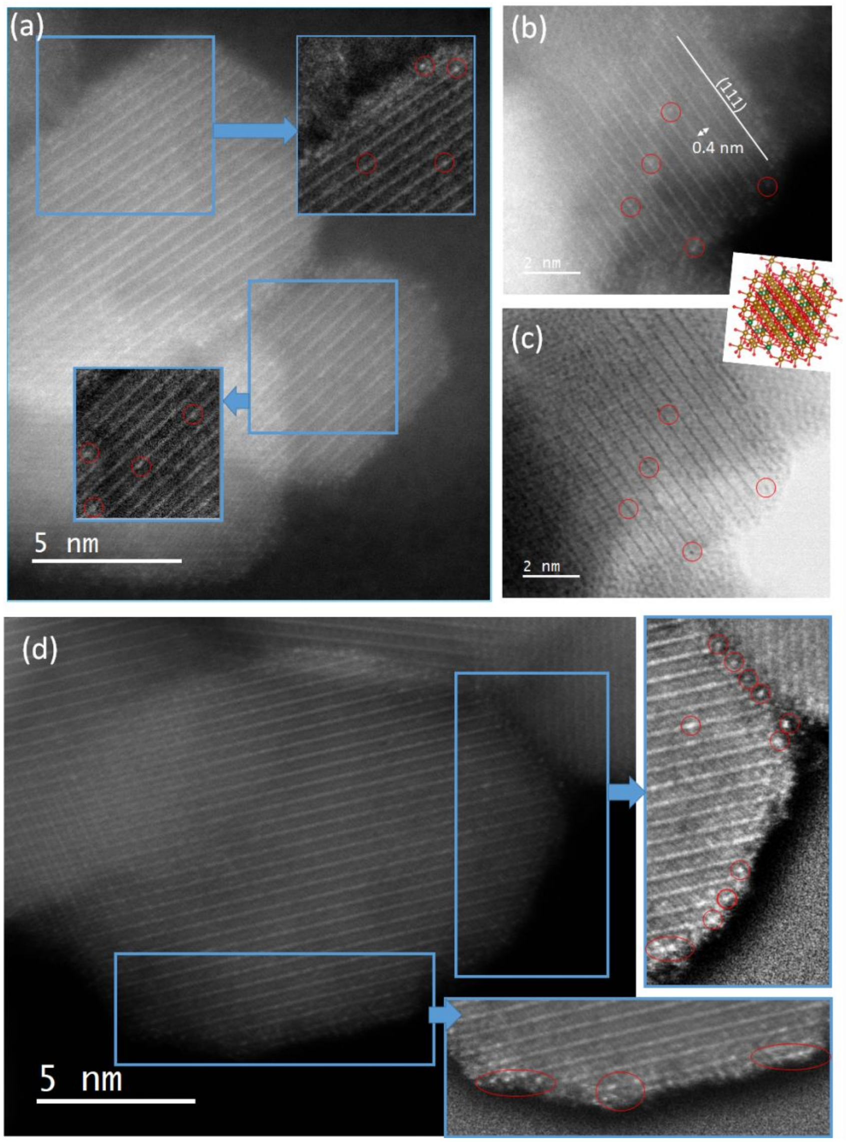

Figure S7. (a,b,c) STEM images of doped magnetite containing 4% Gd as Fe(III). (b) is an HAADF image and the blue boxes indicate the areas where contrasts have been normalized in order to show clearer Gd atoms. The (b) and (c) images are HAADF and annular bright field images of a doped magnetite. (d) HAADF-STEM image of Gd adsorbed on pure nanomagnetite with blue boxes indicating areas where the contrasts have been normalized.

Table S1 Gd(III) doped magnetite solids calculated parameters after Rietveld refinements of the recorded XDR, performed with the BGMN software and Profex user interface.

ID	XDR calculated parameters		Mineral phases detected (% mass)			
	[b]unit cell length (a)	Grain Size magnetite ± 2 (nm)	[a]Magnetite and maghemite ± 2	Goethite ± 2	Green rust ± 2	Amorphous ± 2
**Magnetite**	0.8390 (0.0001)	13	100	-	-	-
**1% Gd**	0.8398 (0.0003)	13	100	-	-	-
**3% Gd**	0.8409 (0.0002)	15	100	-	-	-
**4% Gd**	0.8403 (0.0002)	13	100	-	-	-
**5% Gd**	0.8402 (0.0002)	14	100	-	-	-
**6% Gd**	0.8393 (0.0002)	12	95	5	-	-
**7% Gd**	0.8398 (0.0003)	18	55	42	3	-
**8% Gd**	0.8404 (0.0003)	13	79	16	5	-
**9% Gd**	0.8396 (0.0003)	11	87	11	2	-
**10% Gd**	0.8405 (0.0004)	13	92	8	-	-
**12% Gd**	0.8398 (0.0007)	10	84	-	-	16
**Mag_pH3**	0.8369 (0.0002)	13	100	-	-	-
**Mag_pH5**	0.8400 (0.0003)	13	100	-	-	-

[a] The magnetite reference position is a=8.3958 A (ICDD: 04-005-4319), and for maghemite (cubic) is a=8.336 A (ICSD: 250541).

[b] The measurement error is shown in brackets.

Table S2 ^{57}Fe Mossbauer parameters for magnetite samples recorded at 300 and 77 K. IS is isomer shift relative to αFe; 2ε quadrupole shift and $B_{hf,}$ the hyperfine field.

**Sample**	**T (K)**	**<IS> ± 0.01 (mm s^{-1})**	**<2ε> ± 0.01 (mm s^{-1})**	**<B$_{hf}$> ± 1 (T)**	**% Magnetite ± 4**
Magnetite	300	0.52$_3$	0.01	45.3	100
	77	0.60$_5$	0.02	49.2	100
1% Gd	300	0.51$_9$	0.00	33.1	100
	77	0.60$_7$	0.03	50.1	83
3% Gd	300	0.50$_6$	-0.05	33.2	100
	77	0.64$_2$	0.03	50.1	84
5% Gd	300	0.50$_6$	0.02	36.0	95
	77	0.64$_2$	0.05	50.4	100
7% Gd	300	0.47$_6$	-.07	39.2	80
	77	0.57$_2$	-0.07	49.4	65
9% Gd	300	0.42$_2$	0.09	26.4	52
	77	0.54$_1$	0.12	47.5	51
Mag_pH3	300	0.34$_9$	0.026	42.7	14
	77	0.47$_6$	0.02	51.6	18
Mag_pH5	300	0.41$_2$	-0.00	44.8	47
	77	0.52$_6$	0.06	50.3	43
